# Does endoscopic magnetic resonance imaging challenge EUS?

**DOI:** 10.1097/eus.0000000000000151

**Published:** 2025-12-15

**Authors:** Peng Wu, Wenxin Zhang, Tingting Wang, Lixue Xu, Zhihua Ren, Yi Li, Yang Xia, Jian Zhu, Qi Liu, Shen Pan, Kai Zhang, Siyu Sun

**Affiliations:** 1 Department of Gastroenterology, Endoscopic Center, Shengjing Hospital of China Medical University, Shenyang, Liaoning Province, China; 2 Institute of Health Sciences, Key Laboratory of Precision Diagnosis and Treatment of Gastrointestinal Tumors, China Medical University; Ministry of Education, China Medical University, Shenyang, China; 3 Department of Radiology, Capital Medical University affiliated Beijing Friendship Hospital, Beijing, China; 4 School of Biomedical Engineering & State Key Laboratory of Advanced Medical Materials and Devices, Shanghai Tech University, Shanghai, China; 5 Shanghai Clinical Research and Trial Center, Shanghai, China; 6 Changzhou United Imaging Healthcare Surgical Technology Co., Ltd, Changzhou, China; 7 Department of Clinical Epidemiology, Shengjing Hospital of China Medical University, China Medical University, Shenyang, Liaoning Province, China; 8 Department of General Surgery, Shengjing Hospital of China Medical University, Shenyang, Liaoning Province, China; 9 Shanghai United Imaging Healthcare Advanced Technology Research Institute Co., Ltd., Shanghai, China; 10 Department of Nuclear Medicine, Shengjing Hospital of China Medical University, Shenyang, Liaoning Province, China; 11 Engineering Research Center of Ministry of Education for Minimally Invasive Gastrointestinal Endoscopic Techniques, Shengjing Hospital of China Medical University, Shenyang, Liaoning Province, China.

**Keywords:** Endoscopic magnetic resonance imaging, EUS, Wireless coil technology, Balloon stabilization, Digestive system imaging, Esophageal imaging, Inductive coupling, Image quality assessment

## Abstract

**Background and Objectives:**

EUS is widely used in diagnosing and treating digestive system diseases. However, its diagnostic accuracy is frequently compromised by physiological motion from respiration and cardiac activity, as well as interference from gas. To address these limitations, we developed a wireless endoscopic magnetic resonance imaging (EndoMRI) system with balloon-assisted stabilization technology.

**Methods:**

The system features a novel wireless EndoMRI coil that uses inductive coupling technology, designed to operate through standard endoscopic working channels. An inflatable balloon with an adjustable diameter range of 10–35 mm maintains optimal coil positioning within the digestive tract. System performance was evaluated using *ex vivo* porcine esophageal specimens (*n* = 5) at 5.0T magnetic field strength. Comparative analysis with miniprobe EUS encompassed image quality assessment and histopathological correlation.

**Results:**

The results demonstrated that EndoMRI achieved superior esophageal image stability compared to EUS microprobes (*P* < 0.05). In a thermal injury model, EndoMRI signal continuity showed a strong correlation with histopathological findings of tissue damage, enabling accurate assessment of both lesion depth and severity. The wireless balloon-equipped EndoMRI system provides stable and reliable continuous imaging of *ex vivo* esophagi.

**Conclusions:**

These findings establish EndoMRI as a promising technology in digestive system evaluation.

## INTRODUCTION

Digestive system tumors represent a substantial global health challenge, accounting for approximately 25% of worldwide cancer incidence and one-third of all cancer-related deaths, affecting millions of patients.^[1–3]^ Early detection and treatment are crucial for improving patient outcomes in digestive tumors. The evolution of endoscopic techniques has revolutionized both early detection and minimally invasive treatment, significantly improving prognosis for early-stage lesions.^[4–6]^ Accurate preoperative staging is crucial for optimal treatment selection, as inadequate assessment of lesion extent may necessitate conversion from endoscopic resection to surgical management.^[7–14]^

EUS serves as the primary tool for assessing lesion characteristics and invasion depth in digestive tumors.^[5,6,11–14]^ Despite its widespread adoption, EUS faces limitations including operator dependency, restricted field of view, and susceptibility to gas interference, which collectively compromise image quality and diagnostic accuracy.

Magnetic resonance imaging (MRI) technology continues to advance, providing good tissue contrast, multiplanar imaging capabilities, and relative resistance to gas interference.^[15–18]^ The concept of endoscopic MRI (EndoMRI) emerged to combine endoscopic accessibility with MRI’s advanced diagnostic capabilities.^[19–24]^ However, conventional wired EndoMRI implementations face challenges: radiofrequency (RF) heating risks and restricted maneuverability within endoscopic channels.

We developed an innovative wireless EndoMRI system incorporating balloon-based stabilization and wireless coil technology utilizing inductive coupling. This design eliminates physical cable constraints while ensuring optimal coil positioning within the digestive tract through controlled balloon inflation. The present study evaluates this technology’s image quality compared to standard miniprobe EUS in *ex vivo* esophageal specimens.

## METHODS

**Figure 1:**
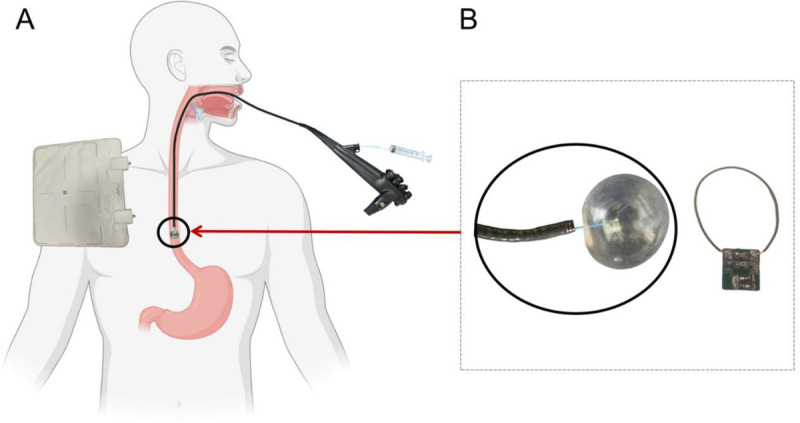
The wireless endoscopic magnetic resonance imaging (EndoMRI) coil system. A, Wireless EndoMRI system comprising an integrated balloon-equipped coil device and external receiving apparatus. The schematic demonstrates the clinical deployment configuration with the endoscopic coil positioned within the digestive tract and wireless signal transmission to the external receiver unit. B, Detailed components of the EndoMRI coil assembly, featuring the imaging coil integrated with a balloon mechanism. The balloon device can be inflated or deflated via syringe injection to optimize tissue contact and imaging stability during the procedure.

The wireless EndoMRI coil was developed in collaboration with Shanghai United Imaging Medical Technology [Figure 1]. The coil assembly can successfully pass through endoscopic working channels and incorporates integrated tuning capacitors with protection diodes that enable automatic detuning during radiofrequency transmission phases. The wireless design eliminates traditional cable connections, using inductive coupling (L1) for signal transmission to the MR system instead. The coil has an external inflatable balloon. The inflatable balloon system provides diameter adjustment ranging from 10.0 to 35.0 mm, enabling adaptation across diverse digestive tract. A miniprobe EUS is used for comparison.

**Figure 2:**
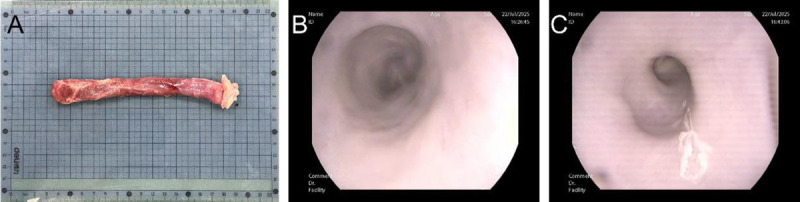
*Ex vivo* porcine esophageal model and endoscopic visualization. A, Fresh *ex vivo* porcine esophagus specimen positioned on measurement grid for experimental preparation. The tissue sample demonstrates the anatomical structure and dimensions suitable for endoscopic evaluation. B, Endoscopic view of the normal intact esophageal model showing the characteristic surface and luminal architecture under standard white light visualization. C, Endoscopic view of the thermal injury esophageal model, demonstrating visible tissue damage and altered appearance consistent with controlled thermal lesion creation for experimental validation of imaging capabilities.

Fresh porcine esophageal specimens (*n* = 5) were obtained from the experimental center and transported on ice. Thermal injury model was established under endoscopy [Figure 2]. Specimens were subsequently examined using standardized imaging protocols on a 5.0T MRI system (Shanghai United Imaging Medical Technology Co., Ltd., Shanghai, China).

Standard imaging protocols encompassed transverse T1-weighted and T2-weighted sequences covering the complete esophageal segments. The wireless EndoMRI coil was tuned to 210.8 MHz. Detailed imaging parameters are provided in Table 1. The wireless coil was carefully inserted into each segment following surface moistening to ensure optimal contact. The balloon component was inflated with air to achieve proper wall apposition and imaging quality. For comparison, miniprobe EUS examinations were performed using standard clinical protocols.

**Table 1:** EndoMRI sequence parameters.

Sequence	TR	TE	Averages	Thickness (mm)	Matrix	FOV (mm)	Voxel size (mm)
tra T1 TSE	766	8.62	4	2	320 × 320	60	0.1 × 0.1
tra T2 TSE	3000	102.2	2	2	320 × 320	60	0.1 × 0.1

FOV: Field of view; TE: Echo time; TR: Repetition time; tra: Transverse.

To assess potential RF heating effects of the wireless EndoMRI coil, temperature measurements were performed before and after MRI examination. For the comparative analysis, each esophageal specimen was divided into four segments for imaging evaluation. EndoMRI acquisition was accomplished by positioning the balloon-stabilized coil and completing the entire imaging sequence in a single scan. In contrast, miniprobe EUS examination of the *ex vivo* specimens required repeated repositioning and multiple acquisitions to achieve adequate visualization of all segments.

Following imaging, esophagi were fixed in 10% formalin solution. Serial 4-μm sections were obtained and stained with hematoxylin and eosin for histological correlation. A radiologist and endoscopist jointly analyzed all images. Histological correlation established the anatomical significance of each layer. Thermal injury sites underwent morphological analysis to assess correspondence between imaging and histological findings. Image quality was independently scored by both observers using the criteria in Table 2.

**Table 2:** Image quality assessment.

Score	Description
4	No distortion or artifacts
3	Some degree of distortion or artifacts
2	Severe distortion or artifacts
1	Unrecognizable image

Statistical analysis utilized R 4.5.1.^[25]^ Intergroup comparisons employed the Wilcoxon signed-rank test. Interobserver agreement was assessed using kappa statistics. *P* < 0.05 was considered statistically significant.

## RESULTS

**Figure 3:**
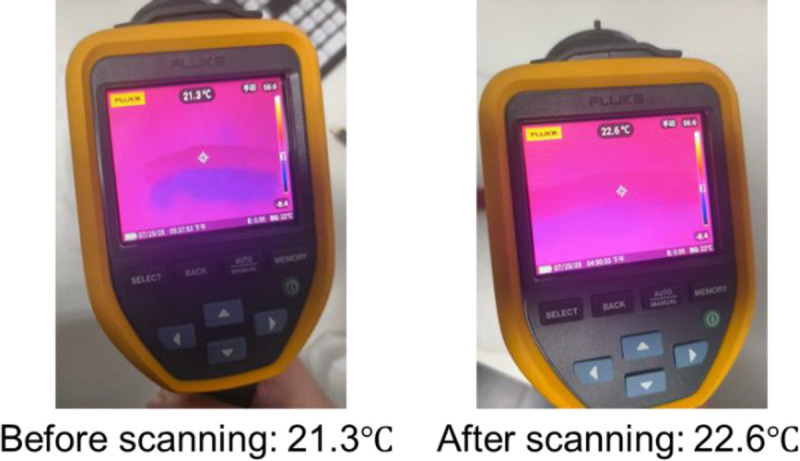
Thermal imaging assessment of wireless coil temperature during scanning procedure. Thermal images captured (A) before scanning showing a baseline temperature of 21.3°C and (B) after completion of the scanning process showing a temperature of 22.6°C. The minimal temperature increase of 1.3°C demonstrates negligible heat accumulation in the wireless coil during the scanning procedure under ambient room temperature conditions.

Temperature monitoring confirmed the safety profile of the wireless design, with maximum temperature elevation remaining below 2°C throughout experiments, well within safe limits for clinical application [Figure 3].

**Figure 4:**
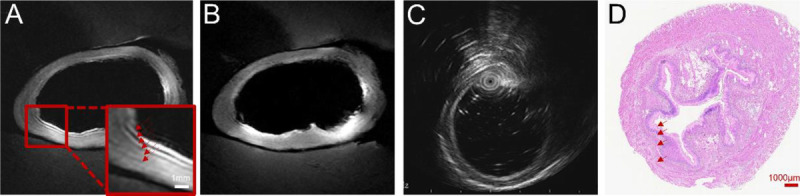
Imaging quality comparison and histological correlation of normal esophageal wall. A, T2-weighted EndoMRI image demonstrating complete and continuous visualization of the entire esophageal wall circumference. The balloon-stabilized coil maintains consistent tissue contact throughout the imaging field. B, T1-weighted EndoMRI image of the same segment confirming stable imaging quality with complete wall visualization across different sequence parameters. C, Miniprobe EUS image showing partial esophageal wall visualization with areas of signal discontinuity due to intraluminal gas interference. D, Corresponding histological section stained with hematoxylin and eosin for anatomical reference. Scale bars represent 1000 μm for histological section.

In normal esophageal segments, the EndoMRI system demonstrated complete and continuous circumferential visualization of the esophageal wall structure during all imaging acquisitions [Figure 4]. The balloon stabilization mechanism maintained optimal coil positioning throughout the imaging process, enabling continuous coverage without requiring repositioning. Both T1-weighted and T2-weighted sequences demonstrated high-quality visualization across the entire field of view. In contrast, miniprobe EUS imaging was significantly compromised by gas interference, necessitating multiple repositioning attempts that resulted in discontinuous wall visualization. Areas of signal loss required repeated probe adjustments to achieve adequate anatomical coverage.

**Figure 5:**
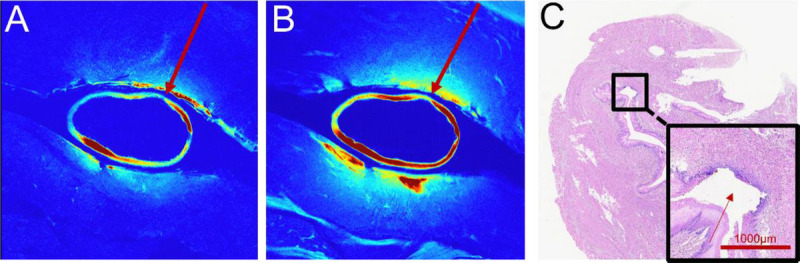
Imaging quality assessment in thermally injured esophageal segments. A, T2-weighted EndoMRI image showing complete and uninterrupted visualization of the thermally injured esophageal wall. Despite tissue alterations from thermal injury, the balloon-equipped coil maintains continuous imaging coverage. B, T1-weighted EndoMRI image confirming consistent imaging quality and complete circumferential visualization of the injured segment. C, Histological section with hematoxylin and eosin staining providing pathological correlation of the thermal injury. The magnified inset shows the extent of tissue damage. Scale bars represent 1000 μm for the histological section.

In thermally injured esophageal segments, the EndoMRI system maintained its imaging stability and completeness despite the presence of tissue alterations [Figure 5]. Both T2-weighted and T1-weighted sequences successfully captured the full extent of thermal injury. Although the EndoMRI system maintained consistent image quality throughout the injured segments, miniprobe EUS examinations encountered additional challenges in these areas. This difference in imaging completeness has important implications for clinical assessment, as continuous visualization is essential for accurate determination of lesion extent and depth.

This study compared image quality between EndoMRI and EUS through rigorous ordinal data analysis. Twenty paired images were independently evaluated by two raters using a standardized 4-point scale (Table 2). Interrater reliability analysis revealed better consistency for EndoMRI (kappa = 0.451, moderate agreement) compared with EUS (kappa = 0.327, fair agreement). EndoMRI demonstrated superior image quality versus EUS (median scores: 3.0 [IQR: 0.625] *vs.* 2.25 [IQR: 0.5]; *P* < 0.05, Wilcoxon signed-rank test; *n* = 20).

## DISCUSSION

This study demonstrates that wireless balloon-equipped EndoMRI provides superior imaging stability and continuous visualization compared to conventional EUS in *ex vivo* esophageal evaluations. The technology addresses limitations of previous EndoMRI approaches through innovative design solutions. Our findings align with emerging evidence supporting MRI’s role in digestive tract evaluation.^[26–30]^

By employing inductive coupling technology, the wireless architecture substantially reduces radiofrequency heating—a major safety concern that has previously limited the clinical translation of wired EndoMRI systems [Figure 3]. Our temperature monitoring confirmed safe operation within acceptable limits, supporting potential clinical translation. EndoMRI provides high-resolution visualization of the multilayered architecture of the esophageal wall, allowing clear differentiation of distinct tissue layers [Figure 4]. The balloon stabilization mechanism proved essential for maintaining consistent image quality. Unlike EUS, which requires continuous manual adjustments to compensate for gas interference and motion, the EndoMRI system achieved stable positioning through controlled balloon inflation.^[13,14,31–34]^ This design enables standardized acquisition protocols that could reduce operator dependency.

The ability to maintain continuous visualization has important clinical implications. Complete image assessment is crucial for accurate staging and evaluation, particularly for early neoplastic lesions where precise depth assessment determines treatment strategy.^[35–37]^ The EndoMRI system’s single-scan efficiency could streamline workflow of endoscopic procedures. The thermal injury model employed in this study demonstrated the EndoMRI system’s capability to visualize pathological tissue alterations while maintaining imaging stability [Figure 5]. The system successfully captured complete visualization of injured segments, with imaging quality remaining consistent despite tissue changes. This capability indicates valuable clinical potential for assessing pathological abnormalities where continuous visualization is essential for accurate diagnosis.

This study has some limitations, primarily due to the *ex vivo* experimental setting. Future research directions include *in vivo* validation studies, development of specialized sequences for tissue characterization, and integration with existing endoscopic platforms. Furthermore, the potential incorporation of functional imaging capabilities, such as diffusion-weighted imaging and perfusion assessment, could substantially enhance diagnostic capabilities beyond conventional EUS.^[38–40]^

In conclusion, the wireless balloon-equipped EndoMRI system demonstrates reliable continuous esophageal imaging with enhanced diagnostic potential. The combination of uninterrupted circumferential visualization, reduced gas susceptibility, and standardized acquisition protocols positions this technology as a promising advancement in digestive imaging. Clinical translation studies are warranted to validate these encouraging *ex vivo* findings. As the technology progresses toward clinical implementation, particularly with the addition of functional imaging modalities, EndoMRI may challenge EUS in comprehensive digestive system evaluation.

## Acknowledgments

We acknowledge Shanghai United Imaging Medical Technology Co., Ltd. For technical assistance with the 5.0T MRI system operation and wireless EndoMRI coil development. We thank the Department of Pathology at Shengjing Hospital for histological processing and analysis support.

## Conflicts of Interest

Siyu Sun is the Editor-in-Chief of the journal. The article was subject to the journal’s standard procedures, with peer review handled independently of this editor and his research groups. Yi Li is affiliated with Changzhou United Imaging Healthcare Surgical Technology companies. Qi Liu is affiliated with Shanghai United Imaging Healthcare companies for the development of the wireless EndoMRI coil system. The companies provided technical support and access to the 5.0T MRI system. All other authors declare no competing financial or nonfinancial interests.

## Ethical Approval

Not applicable.

## Informed Consent

Not applicable.

## Author Contributions

K. Zhang, S. Sun, and S. Pan designed research; P. Wu, W. Zhang, T.Wang, L. Xu, J. Zhu, and Y. Xia conducted research; Z. Ren, Y. Li, and Q. Liu provided essential materials; P. Wu analyzed data; P. Wu, W. Zhang, and T.Wang wrote the paper; K. Zhang and S. Sun had primary responsibility for the final content. All authors read and approved the final manuscript.

## Data Availability Statement

The datasets generated and analyzed during the current study are available from the corresponding author upon reasonable request. Raw MRI data and histological images are stored in the institutional repository and can be accessed with appropriate data transfer agreements.
